# A Survey of Deep Learning-Based Pedestrian Trajectory Prediction: Challenges and Solutions

**DOI:** 10.3390/s25030957

**Published:** 2025-02-05

**Authors:** Jiaming Jiang, Kai Yan, Xindong Xia, Biao Yang

**Affiliations:** Wang Zheng Institute of Microelectronics, Changzhou University, Changzhou 213000, China; czujjm@cczu.edu.cn (J.J.); s22060809035@smail.cczu.edu.cn (K.Y.); s23060854038@smail.cczu.edu.cn (X.X.)

**Keywords:** trajectory prediction, motion uncertainty, interaction modeling, scene semantic understanding, interpretability

## Abstract

Pedestrian trajectory prediction is widely used in various applications, such as intelligent transportation systems, autonomous driving, and social robotics. Precisely forecasting surrounding pedestrians’ future trajectories can assist intelligent agents in achieving better motion planning. Currently, deep learning-based trajectory prediction methods have demonstrated superior prediction performance to traditional approaches by learning from trajectory data. However, these methods still face many challenges in improving prediction accuracy, efficiency, and reliability. In this survey, we research the main challenges in deep learning-based pedestrian trajectory prediction methods and study this problem and its solutions through literature collection and analysis. Specifically, we first investigate and analyze the existing literature and surveys on pedestrian trajectory prediction. On this basis, we summarize several main challenges faced by deep learning-based pedestrian trajectory prediction, including motion uncertainty, interaction modeling, scene understanding, data-related issues, and the interpretability of prediction models. We then summarize solutions for each challenge. Subsequently, we introduce mainstream trajectory prediction datasets and analyze the state-of-the-art (SOTA) results reported on them. Finally, we discuss potential research prospects in trajectory prediction, aiming to promote the trajectory prediction community.

## 1. Introduction

With the development of artificial intelligence, pedestrian trajectory prediction has garnered attention in various applications, such as intelligent transportation systems, autonomous driving, security monitoring, and sports analysis. For example, by accurately predicting the surrounding pedestrians’ future trajectories, autonomous vehicles can optimize path planning and adjust their behaviors in real-time to avoid pedestrian–vehicle conflict. This predictive capability is especially vital in urban environments, where pedestrian traffic is dense and interactions between pedestrians, vehicles, and cyclists are frequent and complex. Moreover, real-time pedestrian tracking and prediction systems can alert pedestrians to potential hazards, such as oncoming vehicles or obstacles, thereby enabling them to make safer decisions. Beyond safety, pedestrian trajectory prediction also optimizes traffic flow. By predicting pedestrian movements, traffic management systems can dynamically adjust traffic signals, pedestrian crossings, and vehicle routes to minimize congestion and delays. This not only enhances the overall efficiency of transportation networks but also improves the quality of urban life by reducing commuting times and stress.

Pedestrian trajectory prediction aims to forecast pedestrians’ future trajectories based on their historical trajectories and the environmental context. Traditional trajectory prediction methods are primarily divided into kinematic-based and machine-learning-based methods. The former contains constant velocity, acceleration, and turn rate models [1,2,3]. Despite the benefits of easy implementation and efficient computation, kinematic models need to adequately account for the uncertainty in the current state during prediction. In contrast, machine learning-based methods resort to Support Vector Machine (SVM) [4] and Dynamic Bayesian Networks (DBN) [5] to model pedestrians’ motion patterns. However, they are less effective at capturing complex motion patterns. Moreover, careful feature engineering requires significant domain knowledge and expertise.

Pedestrian trajectory prediction is a time series analysis issue. With the rise of deep learning, Recurrent Neural Networks (RNN) [6], Convolutional Neural Networks (CNN) [7], Graph Neural Networks (GNN) [8], and Transformers [9] have been used for pedestrian trajectory prediction due to their powerful data-driven learning capabilities. Therefore, a comprehensive survey of pedestrian trajectory prediction has become increasingly necessary.

The survey in ref. [10] reviews and analyzes pedestrian trajectory prediction methods and proposes a taxonomy that categorizes existing methods based on the motion modeling approach and level of contextual information used. Specifically, based on the motion modeling approach, the existing prediction methods are summarized into three categories: physics-based, pattern-based, and planning-based. On the other hand, the authors define contextual cues as all relevant internal and external stimuli that influence motion behavior and categorize the existing prediction methods based on their relation to the target agent, other agents in the scene, and properties of the static environment. However, this survey does not explicitly discuss the challenges of pedestrian trajectory prediction.

The survey in ref. [11] targets intention and trajectory prediction in autonomous vehicles (AV). It critically reviews the ability of various models’ building blocks to model the prediction task’s social, temporal, and generative dimensions. This survey mentions that prediction in the AV domain faces challenges due to the dynamic, multi-agent, and stochastic characteristics of the driving environment. However, it does not list and analyze the solutions to the challenges described.

As human behavior is naturally multimodal and uncertain, the survey [12] primarily focuses on various frameworks for multimodal trajectory prediction. Ref. [13] details the most utilized deep learning-based methods for pedestrian trajectory prediction, which are classified according to the network architectures, including RNN (typically the long short-term memory (LSTM)), CNN, and Generative Adversarial Networks (GAN). Another review [14] compares deep learning models with classical knowledge-based models widely used to simulate pedestrian dynamics. It provides a comprehensive literature review of both approaches and explores technical and application-oriented differences. The investigations show that deep learning algorithms accurately predict local trajectories. Nevertheless, the ability of deep learning algorithms for large-scale simulation and the description of collective dynamics remains to be demonstrated. To sum up, refs. [13,14] only classify and analyze the deep learning-based pedestrian trajectory prediction methods based on the network architectures and do not categorize these methods according to challenges that can be solved.

To promote the further development of deep learning-based pedestrian trajectory prediction and improve its practicability, this paper researches the challenges in deep learning-based pedestrian trajectory prediction methods and their current solutions. Through literature collection and review, we analyze the main challenges to be solved in the field of pedestrian trajectory prediction, including motion uncertainty, interaction modeling, scene understanding, data-related issues, and interpretability of prediction models. In response to these challenges, we study and summarize possible solutions to different challenges and review the effectiveness of some advanced solutions. The contributions are summarized as follows:

(1) We summarize the challenges faced by deep learning-based pedestrian trajectory prediction from the aspects of the pedestrians themselves, the objective scene where pedestrians located, and the prediction model itself. Specifically, we categorize the challenges as motion uncertainty, interaction modeling, scene understanding, data-related issues, and the interpretability of prediction models.
(2) We conduct a detailed exploration of solutions corresponding to these challenges and provide a comprehensive summary.
(3) We survey the publicly available trajectory prediction datasets and report the State-Of-The-Art (SOTA) results on them with in-depth analysis, highlighting effective methods in trajectory prediction.

The remaining sections of this work are organized as follows: Section 2 introduces the preliminary knowledge of trajectory prediction; Section 3 summarizes the challenges and potential solutions; Section 4 summarizes publicly available datasets, evaluation metrics, and SOTA results; Section 5 presents the conclusion and discusses the future directions that can facilitate pedestrian trajectory prediction.

## 2. Preliminary Knowledge

### 2.1. Problem Formulation

Pedestrian trajectory prediction is a time series analysis issue. The essence of trajectory prediction is to predict pedestrians’ future locations based on their historical trajectories. A trajectory of a pedestrian *i* in trajectory prediction is defined as a sequence of two-dimensional (2D) real-world or pixel coordinates: {$X_{i}^{T},\;Y_{i}^{\tau }$}, where $X_{i}^{T}\;$= {$X_{i}^{t}$| t ∈ [1, *T*_obs_]} is the observed trajectory with *T*_obs_ time steps; $Y_{i}^{\tau }\;$= {$Y_{i}^{t}$| t ∈ [*T*_obs_, *T*_obs_ + *T*_pred_]} is the ground truth of the future path with *T*_pred_ time steps; and *i* is the index among *N* pedestrians in a scene *i* ∈ [1, *N*]. Both $X_{i}^{t}$ and $Y_{i}^{t}$ contain 2D coordinates.

The goal of trajectory prediction is to optimize a model *f*_TP_ to predict *K* future trajectories ${\hat{Y}}_{i,\;K}^{\tau }$ = {${\hat{Y}}_{i,\;k}^{\tau }$|*k* = 1, …, *K*} using observed information $X_{i}^{T}$, $X_{1:N\backslash i}^{T}$, *S* as inputs:

(1) $${\hat{Y}}_{i,\;K}^{\tau }=f_{\mathrm{TP}}\;(X_{i}^{T},\;X_{1:N\backslash i}^{T},S)$$

where $X_{1:N\backslash i}^{T}$ is the set of pedestrian *i*’s neighbors’ observed trajectories; and *S* is the scene information, which can be the LIDAR data, high-definition (HD) maps, scene images, etc. When *K* = 1, where only a single prediction is allowed for each pedestrian, the task is deterministic trajectory prediction and expects a minimum prediction error compared to $Y_{i}^{\tau }$. Otherwise, it becomes Multimodal Trajectory Prediction (MTP) and aims to predict a distribution of all acceptable future trajectories.

### 2.2. Existing Challenges

While deep learning has significantly improved trajectory prediction performance, it still faces several challenges. The surveys in [10,11] have mentioned some challenges to pedestrian trajectory prediction, such as semantic scene understanding, multi-agent social interaction, uncertainty, and multimodality of agents’ behaviors. On this basis, by further sorting out and analyzing existing literature, we provide a more comprehensive summary of the main challenges faced by pedestrian trajectory prediction. First, as the objects to be predicted, pedestrians’ subjective behaviors and characteristics have a significant impact on the trajectory prediction results, including the uncertainty of pedestrian movement and the interaction between multiple pedestrians. Secondly, in practical applications, the objective scenes in which pedestrians are located can affect their walking trajectories, so a good understanding of the scenes is required to make accurate predictions. Trajectory prediction also requires obtaining accurate and reliable scene data and dealing with data with defects. Finally, as for deep learning techniques and their models themselves, interpretability is required to improve models’ reliability and make them suitable for security-related applications.

**Figure 1:**
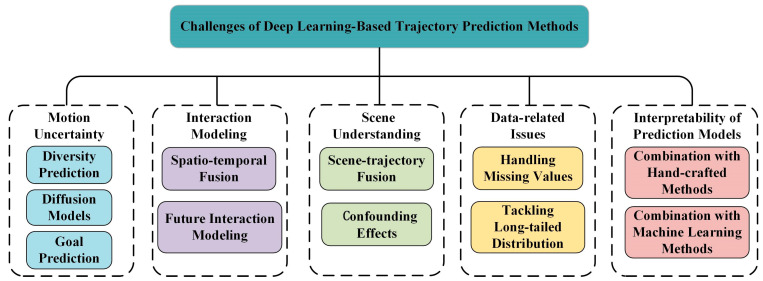
Overview of the challenges and corresponding solutions.

Based on the above analysis, it can be summarized that there are five challenges in deep learning-based pedestrian trajectory prediction: (1) **Motion uncertainty**: The future motion of pedestrians involves uncertainty. Pedestrians may adhere to social rules and travel along roads, or they may choose to cross roads to reach other destinations. Therefore, handling motion uncertainty and making reasonable predictions is challenging. (2) **Interaction modeling**: Modeling interactions between pedestrians is challenging. Others may influence the pedestrian’s decision-making process. It is necessary to consider interactive factors to enhance prediction accuracy thoroughly. (3) **Scene understanding**: Pedestrians need to navigate around obstacles during their movements, so a comprehensive understanding of the scene is crucial for more accurate trajectory prediction. (4) **Data-related issues**: Trajectory data exhibit missing values and long-tailed distributions, fundamentally affecting prediction accuracy. (5) **Interpretability**: Deep learning-based pedestrian trajectory prediction lacks interpretability, reducing the model’s reliability. The overview of the challenges and corresponding solutions is shown in Figure 1.

## 3. Solutions to Challenges

This section summarizes the solutions to the challenges mentioned above and provides a comprehensive summary.

### 3.1. Motion Uncertainty

The motion of pedestrians is inherently stochastic and uncertain. The uncertainty is influenced by various factors, making accurate predictions of future trajectories challenging. To fully account for uncertainty in trajectory prediction, diversity prediction is a common approach that predicts multimodal trajectories. Furthermore, the diffusion model has proven highly effective in capturing the uncertainties in trajectory prediction, rendering it a frequently used solution to mitigate motion uncertainty. The goals of agents reflect their ultimate intentions. The predicted goals can serve as conditional information for trajectory prediction, thereby addressing motion uncertainty to some extent. This section introduces motion uncertainty solutions from three perspectives: diversity prediction, diffusion models, and goal prediction.

#### 3.1.1. Diversity Prediction

**Figure 2:**
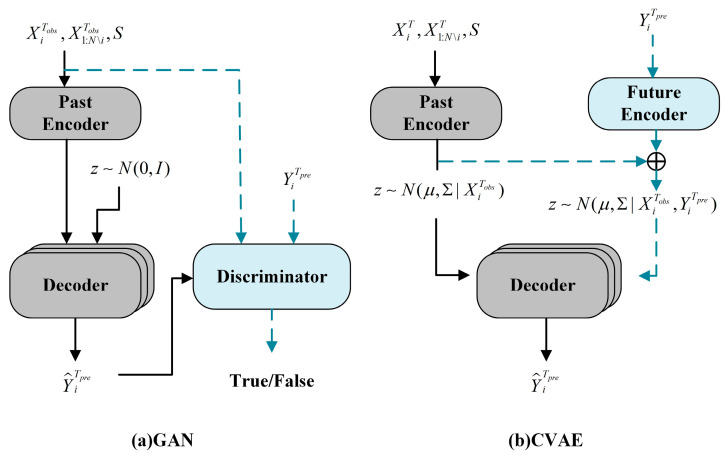
Diagrams of GAN and CVAE structures.

Recent studies have focused on the diversity prediction of trajectories, with many adopting Generative Adversarial Networks (GANs) [15]. Figure 2a illustrates that GAN consists of a generator and a discriminator. The former generates diverse trajectories by introducing random noise. The latter distinguishes the trajectories predicted by the generator from the observed ones, making the generated trajectories more realistic and diverse. Kosaraju et al. [16] introduced a graph-based GAN dubbed Social-BiGAT, establishing a reversible mapping between the generated trajectories and latent variables of pedestrian movements. This encourages the model to generate trajectories that can adapt to multi-modal distributions. Gupta et al. [17] proposed a diverse loss to encourage the generator to produce various trajectories. However, the loss may lead to mode collapse. Therefore, Amirian et al. [18], drawing inspiration from Info-GAN [19], incorporated latent encoding into the original random noise to enhance trajectory prediction. Except for mode collapse, GANs may generate out-of-distribution (OOD) samples due to the discontinuity of the trajectory distribution manifold. Dendorfer et al. [20] proposed that MG-GAN dynamically selects from multiple generators for prediction to address this issue. Recently, Wang et al. [21] proposed a Sequence Entropy Energy Model (SEEM). SEEM utilized maximum sequence entropy as the loss for the generator, ensuring that the generated trajectories encompass all modes of pedestrian behaviors. Additionally, SEEM introduced zero-centered potential energy regularization to enhance training stability.

The Conditional Variational Auto-encoder (CVAE) [22] is another multi-modal trajectory prediction framework. As shown in Figure 2b, CVAE involves mapping the input data to a latent variable distribution through an encoder. Afterward, multiple samples of latent variables are drawn from the latent distribution as conditions. The decoder generates multiple possible trajectories, maximizing the lower-bound loss to achieve multi-modality in generation. Due to conditional information, CVAE provides better control and enhancement over the latent distribution. Chen et al. [23] employed self-supervised contrastive learning to categorize latent distributions, thereby predicting personalized multi-modal trajectories. Xu et al. [24] introduced Social-VAE for pedestrian trajectory prediction. They employed a time-wise VAE to generate as many trajectory samples as possible from the predicted distribution, inferring the distribution of the pedestrians’ future locations. Final Position Clustering (FPC) was used to enhance prediction diversity. However, CVAE cannot generate as realistic outputs as GAN. Moreover, CVAE encounters the OOD issue. To tackle this issue, Guo et al. [25] applied a symmetric cross-entropy loss, which combined the forward and reverse cross-entropy between the predicted and the ground truth distributions. The reverse cross-entropy was used to penalize OOD predictions. Furthermore, Zhou et al. [26] combined CVAE and GAN for multi-modal trajectory prediction, alleviating the mode collapse issue GAN caused and addressing the problem of requiring additional and hard-to-define losses in CVAE.

#### 3.1.2. Diffusion Models

**Figure 3:**
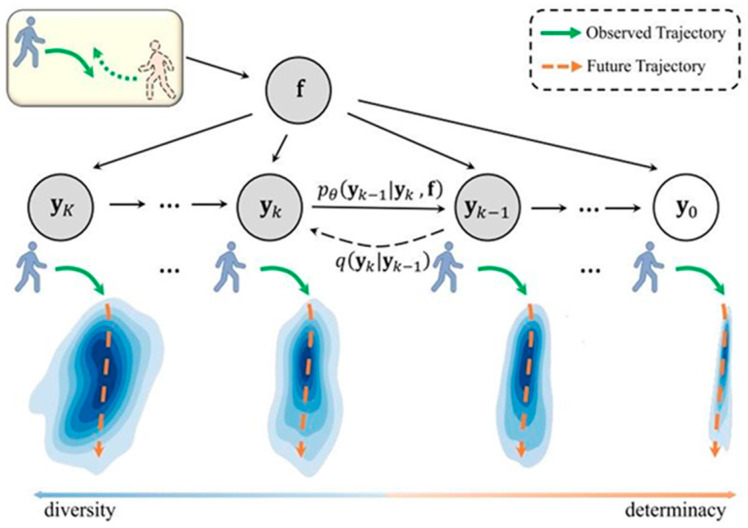
Illustration of the reverse diffusion process of pedestrian motion uncertainty variation [27]. Given the initial ambiguous region **y***_K_* under the noise distribution and the desired trajectory **y**_0_ under the data distribution, we define the diffusion process as (**y**_0_, **y**_1_, · · ·, **y***_K_*), where *K* is the maximum number of diffusion steps. This process aims to gradually add the indeterminacy until the ground truth trajectory is corrupted into a noisy walkable region. On the contrary, we learn the reverse process as (**y***_K_*, **y***_K_*_−1_, · · ·, **y**_0_) to gradually reduce the indeterminacy from **y***_K_* to generate the trajectories. Both diffusion and reverse diffusion processes are formulated by a Markov chain with Gaussian transitions.

To solve the inherent shortcomings of GAN and CVAE in trajectory generation, the diffusion model is proposed to learn a parameterized Markov chain that progressively evolves from an initial shared distribution to a specific target data distribution through iterations. As illustrated in Figure 3, Gu et al. [27] proposed a Transformer-based MID, achieving trajectory prediction through a reverse process of motion uncertainty diffusion. They encoded historical trajectories and social interactions into state embeddings using a spatiotemporal GNN, which served as conditions for the Markov chain. These conditions guided the learning of the reverse diffusion process to generate future trajectories. Chen et al. [28] considered trajectories’ geometric equivariance, proposing combining an equivariant Transformer with a denoising diffusion to generate future trajectories with historical information and random Gaussian noise.

However, the computational cost of the diffusion model is high because it requires multiple denoising steps to ensure representational capacity. Mao et al. [29] applied the Leapfrog Initializer instead of diffusion models for trajectory prediction to address this issue. This approach allowed the model to skip numerous denoising steps and directly learn a rich multi-modal distribution of future trajectories, significantly accelerating the inference speed. Additionally, Liu et al. [30] reconstructed future trajectories using a multi-parameter bivariate Gaussian distribution to perform diffusion modeling. It avoided the complex process of reverse diffusion calculations and separated individual uncertainties from the complex multi-agent motion modeling, thereby more effectively addressing uncertainty issues in trajectory prediction.

#### 3.1.3. Goal Prediction

Recent research has focused on the ultimate goal of the agents, aiming to augment trajectory prediction methods. Mangalam et al. [31] proposed a long-term trajectory prediction method dubbed Y-net, which divided agents’ uncertainty into epistemic and aleatoric uncertainties. They modeled epistemic and aleatoric uncertainties by predicting the goals and waypoints. Lee et al. [32] introduced MUSE-VAE, which aimed to predict long-term and short-term goals by jointly learning the feature representations of the environment and agent motion in a macroscopic stage. In the microscopic stage, it utilized fine-grained spatiotemporal representations to predict the trajectories of individual agents, thus comprehensively addressing uncertainty at two different granularity levels. Chiara et al. [33] utilized historical trajectories and scene semantic information to forecast future goals. Using a recurrent attention mechanism, they enhanced prediction accuracy by incorporating goal information into the forward network. While the methods mentioned above learned goals from the historical trajectories and the scene, they overlooked the impact of interactions on the goals of agents. Choi et al. [34] predicted the goals of target agents by observing traffic scenarios and interactions. They established causal relationships between goals and driver behaviors to reduce uncertainty and achieve more accurate predictions. Zhang et al. [35] introduced a goal-based trajectory prediction method, ForceFormer, which integrated social forces [36,37] into a Transformer-based generative model. It guided pedestrian movement by leveraging the driving forces from the goals and modeled interactions through the environment’s and neighbors’ repulsive forces. Although the above methods have achieved significant success in the trajectory prediction community, accurately predicting destinations in unfamiliar environments is still challenging.

#### 3.1.4. Summary

In order to compare the results of different methods on motion uncertainty and trajectory prediction, Table 1 shows the prediction results of five methods (or models) on the Stanford Drone Dataset (SDD) [38]. The average displacement error (ADE) and final displacement error (FDE), which are defined in formula (2) below, are used as metrics to evaluate the different methods. As shown in Table 1, TDOR [25] achieves better performance than SocialVAE [24] in the diversity prediction of trajectories due to the use of reverse cross-entropy to penalize OOD predictions. MUSE-VAE [32] performs better than Goal-SAR [33] due to the ability to comprehensively address uncertainty at macroscopic and microscopic levels. TDOR [25] and MUSE-VAE [32] achieve the best performance among all five methods, which means diversity prediction methods and goal prediction methods are able to obtain promising results. The result of MID [27] indicates that the methods that adopt diffusion models can achieve compromise performance.

Table 2 summarizes the aforementioned approaches for mitigating the effects of motion uncertainty. Motion uncertainty is a critical challenge in pedestrian trajectory prediction despite the great success achieved by generative models such as CVAE, GAN, and the diffusion model. In contrast to early solutions that generate multi-modal trajectories with random noises, current studies focus on forecasting pedestrians’ intentions, such as goals and waypoints, which are more reasonable to mitigate motion uncertainty. However, uncertainty still exists in predicted intentions, affecting the prediction of multi-modal trajectories. Therefore, it is necessary to forecast potential intentions by better understanding the pedestrian itself and the environmental context.

**Suggestions:** In order to solve the problem of motion uncertainty in trajectory prediction, we propose to combine pedestrian historical motion laws, scene context and typical pedestrian movement patterns in the scene to better explore the real intention of pedestrians. In addition, since the motion uncertainty comes from the pedestrian’s own uncertainty, it cannot be suppressed entirely. We propose sorting the multimodal trajectories predicted for pedestrians and selecting the appropriate trajectories for the specific downstream tasks.

**Possible limitations:** Scene context understanding requires additional computational resources overhead, which may affect the real-time processing of pedestrian trajectory prediction. To solve this problem, more powerful hardware devices and lighter scene-understanding algorithms are needed.

### 3.2. Interaction Modeling

Complex interactions exist between pedestrians in crowded and dynamically changing scenarios, significantly impacting the prediction of future trajectories, making interaction modeling a crucial aspect of pedestrian trajectory prediction.

Prevalent methods for interaction modeling comprise social pooling layers [39,40], attention mechanisms [41,42], and graph neural networks [43,44,45,46]. A social attention module was used to aggregate neighbors’ interactions based on the correlation between pedestrians’ moving directions and future trajectories [40]. Yang et al. [41] introduced the social graph attention and pseudo-oracle predictor to capture pedestrians’ social interactions and intent states, ameliorating trajectory prediction performance. A collision-aware Graph Transformer [46] was introduced to capture the complex social interactions between traffic-agents. Following that, an additional interaction prediction task that could predict the interaction probabilities between agents was proposed to mitigate the over-smoothing issue of the Graph Transformer via a multi-task learning strategy. However, relying solely on these methods is still insufficient for fully addressing the interaction between pedestrians. The interaction between pedestrians is a dynamic process that spans time and space. It involves the exchange of information and the adaptation of behavior over time. The fusion of spatiotemporal features is significant for modeling interactions between pedestrians. Considering interactions based solely on observed trajectories is not comprehensive, as it cannot guarantee future interactions of trajectories. Therefore, for the challenge of multi-agent interaction modeling, the research can be divided into two aspects: spatiotemporal fusion and future interaction modeling.

#### 3.2.1. Spatiotemporal Fusion

**Figure 4:**
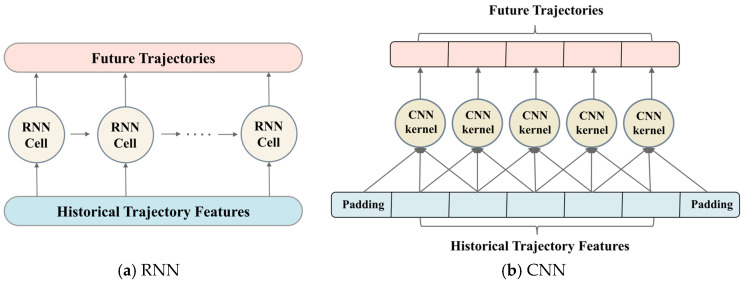
Illustration of Recurrent Neural Network (RNN) and Convolutional Neural Network (CNN) for trajectory prediction [52].

Recently, RNN (typically the LSTM) and CNN have been widely used in trajectory prediction methods. RNN is designed to handle temporal information. It stores information of the previous time steps and utilizes hidden states together with the input to determine the output, as shown in Figure 4a. Choi et al. [47] use an LSTM to model motion information for all pedestrians and use an MLP (multilayer perceptron) to map the location of each pedestrian to a high-dimensional feature space to consider the positional relationship between two pedestrians. Then, weights are assigned to the motion features of all pedestrians based on their positional relationship to the target for location displacement prediction. Through convolutional and pooling layers, CNN extracts local and then global features from the input data. The fully connected layer integrates the extracted features and performs classification or regression tasks. The general methods using CNN to process trajectory information are shown in Figure 4b. Xu et al. [48] proposed a Dynamic-Group-aware Network (DynGroupNet), which utilized dynamic multi-scale hypergraphs to represent pedestrian interactions. It captured the dynamic interactions between pedestrians at different time steps through topological inference, neural message passing, and dynamic embedding evolution of multi-scale hypergraphs. Wang et al. [49] employed a different approach by creating a global spatial tensor through a bird’s-eye view representation, embedding the features of the corresponding time steps to preserve the spatial relationships among agents. Subsequently, they utilized a 3D-CNN to capture the temporal dependencies in the trajectory sequences and employed the Social Recurrent Mechanism (SRM) to model pedestrian interaction. Although existing RNN or CNN-based models have demonstrated high performance in short-term prediction, they still face scalability limitations when dealing with long-term sequences.

**Figure 5:**
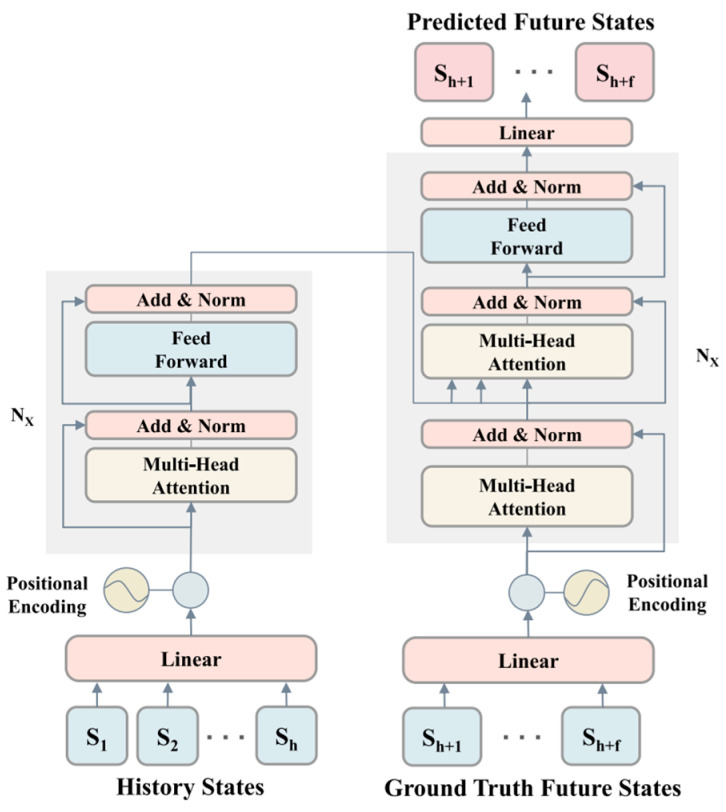
Illustration of Transformer for trajectory prediction [52].

Since Transformers possess powerful spatial and long-term temporal modeling capabilities, they are promising for handling dynamic interactions. The typical application of Transformer for trajectory prediction task is shown in Figure 5. Yuan et al. [50] extracted trajectory features chronologically and fed them into a Transformer to learn spatiotemporal interaction embedding. Yang et al. [51] leveraged GNNs and Transformers to model spatial and temporal dependencies. A Temporal Convolutional Network (TCN) was proposed to integrate spatiotemporal features to consider dynamic interactions between pedestrians comprehensively. However, all these methods ignore the influence of future interaction behavior on future trajectory prediction, which may lead to sub-optimal trajectory generation performance.

#### 3.2.2. Future Interaction Modeling

To consider future interaction modeling, Amirloo et al. [53] modeled interactions between pedestrians using the self-attention mechanism of the Transformer. In the decoding phase, they considered the future states of pedestrians in an auto-regressive manner to avoid conflicts between predicted trajectories. Zhou et al. [54] introduced a novel interaction modeling method, CSR, which differed from other methods by modeling agent interactions after prediction. It extracted features separately from historical trajectories and predicted future trajectories, fusing them into a global motion feature. These motion features were then passed to a social non-local pooling layer [55] to obtain the interaction features. Finally, the interaction features were used to refine the predicted trajectories to avoid unreasonable conflicts. However, due to the uncertainty of the future motion of pedestrians, future interaction modeling between pedestrians is also very complex and challenging.

#### 3.2.3. Summary

The relevant research on interaction modeling is summarized in Table 3. Modeling complex interactions among pedestrians is another challenge for precise trajectory prediction. Traditional studies like social pooling layers, attention mechanisms, and GNNs resort to data-driven methods for interaction modeling. Current studies leverage pedestrians’ future interactions through prediction to improve trajectory prediction performance. However, pedestrian interaction is complicated, and it is challenging to establish an accurate interaction model using only pedestrians’ motion data.

**Suggestions:** To solve the interaction modeling problem in trajectory prediction, we suggest considering the psychological state of pedestrians and the scene layout to build a better pedestrian interaction model. In addition, considering that the future movement trajectory of pedestrians is more affected by the future interaction behavior, we suggest that the future interaction behavior of pedestrians should be modeled to improve the trajectory prediction performance.

**Possible limitations:** Most of the current pedestrian trajectory prediction datasets are obtained from the bird’s eye view, and it is difficult to obtain detailed descriptions of the faces and limbs of pedestrians, so it is difficult to accurately capture the psychological state of pedestrians. In addition, due to the uncertainty of the future trajectory of pedestrians, the uncertainty of future interaction behavior will be further amplified, and how to accurately model future interaction behavior is a possible limitation.

### 3.3. Scene Understanding

Scene semantic information plays a significant role in trajectory prediction and is often used as input to enhance prediction accuracy. In real-world scenarios, the environment is typically complex, with elements such as flower beds, trees, lawns, sidewalks, and traffic signals on the road. These elements impact the motion of pedestrians. Therefore, in trajectory prediction, scene semantic understanding requires recognizing diverse information within the scene. Additionally, effectively fusing semantic information with trajectory data further enhances the model’s accurate understanding of the scene. In addition, scene understanding necessitates an in-depth exploration of the causal relationships between the scene and trajectories due to the confounding effects of the environment. Consequently, this survey introduces scene semantic understanding from two perspectives: scene-trajectory fusion and confounding effects.

#### 3.3.1. Scene-Trajectory Fusion

The semantic information of the scene can be obtained through semantic segmentation [56,57,58]. He et al. [59] used a lightweight semantic segmentation network, ENet [56], to extract scene information. Then, the scene and trajectory information were fused through in-verse reinforcement learning. Additionally, Yang et al. [60] introduced SegFormer [61] to extract scene semantic information, gradually fusing scene and trajectory information at different granularity levels through an efficient scene fusion transformer. While semantic segmentation can effectively understand complex environments, it comes with a high computational cost.

Therefore, some methods directly extracted visual features from scenes and then fused these features with pedestrians’ motion features. Peng et al. [62] utilized CNNs for feature extraction from the scene, and the fusion was achieved using a customized attention mechanism. Xu et al. [63] applied a CNN to extract features from the scene and fused scene and trajectory features with a dual attention mechanism. This approach allowed for a comprehensive consideration of the social features exhibited by pedestrians and the physical environmental constraints, thus generating plausible trajectories. However, pedestrian trajectories are dynamic, so the interaction between trajectories and scenes is also dynamic. The above methods ignore the dynamic influence of trajectory and scene, and the redundant scene information may affect the trajectory prediction accuracy.

#### 3.3.2. Confounding Effects

**Figure 6:**
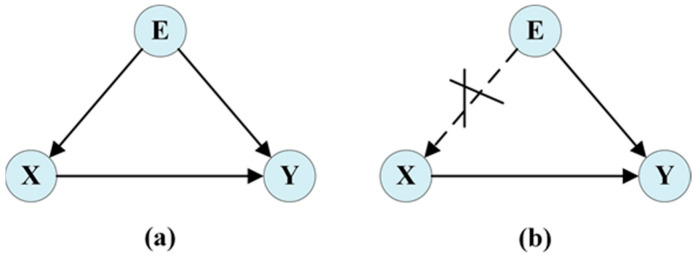
Causal diagram of environmental interactions, historical trajectories, and future trajectories [64]. (**a**): Factual causal graph. (**b**): Counterfactual intervention.

Except for neglecting dynamic trajectory-scene interaction, the bias between the training environment and the practical one is another challenge in scene understanding. Such a bias makes the interaction with the environment become a negative con-founder. Chen et al. [64] pointed out that environmental bias can lead to incorrect predictions in a new environment. To address this, they constructed a causal graph between environmental interactions *E* (social interactions and physical environment interactions), historical trajectories *X*, and future trajectories *Y*, with arrows indicating causal relationships. As shown in Figure 6a, *E* → (*X*, *Y*) indicates that the environment influences pedestrians’ historical and future motion trajectories. According to causal inference [65], when variable E simultaneously affects two variables, *X* and *Y*, it becomes a confounding factor in the causal analysis of these two variables. For example, if right-turn trajectories are frequently associated with intersections during training, the prediction of right-turn trajectories during actual applications may be incorrectly attributed to intersections. The true causal relationship behind historical trajectories may be overlooked. Therefore, to address this issue, they applied counterfactual analysis to trajectory prediction, using the association between the environment and historical trajectories to mitigate the adverse effects of environmental bias (as shown in Figure 6b). Ge et al. [66] and Lian et al. [67] also constructed causal models for environmental interactions of historical and future trajectories to analyze their causal relationships. They used social cross-attention mechanisms and Do-calculus, respectively, to eliminate the confounding effects of the environment. Causal analysis can reveal the factors that affect trajectory prediction in the scene, so it has a significant role in understanding the scene and improving the accuracy of trajectory prediction.

#### 3.3.3. Summary

The relevant methods for scene understanding are summarized in Table 4. Scene understanding is an essential factor in achieving accurate and reliable trajectory prediction. Most former works used CNN or semantic segmentation networks to extract scene information from the scene and then fuse scene and trajectory information through different strategies, such as the attention mechanism. Regarding the confounding effects, the causal model has eliminated the interference caused by environmental differences, thereby improving the model’s performance in practical scenarios. This direction holds significant research potential and can be further explored.

**Suggestions:** In the traditional fusion of scene and trajectory information, the scene and trajectory are regarded as independent components. However, the scene will affect the pedestrian trajectory. Therefore, studying the interaction between scene and trajectory is helpful for better scene understanding. The interaction between multiple trajectories and the scene is more beneficial for reliable scene understanding.

**Possible limitations:** Inverse reinforcement learning provides a way to explore the interaction between a scene and trajectory, but currently, it can only explore the interaction between a single trajectory and scene. Furthermore, understanding scene information requires significant computational resources. Therefore, proposing lightweight network models to achieve efficient scene understanding is also a highly regarded direction. It holds the potential to make significant breakthroughs in trajectory prediction.

### 3.4. Data-Related Issues

Deep learning-based trajectory prediction methods are data-driven methods highly dependent on the reliability and accuracy of trajectory data. However, existing trajectory prediction methods pay little attention to data-related issues. On the one hand, trajectory data may have missing values due to occlusion, range limitation, and sensor failure during acquisition. On the other hand, the trajectory data are unbalanced and have an inherent long-tailed distribution. Therefore, we categorize data-related issues into two aspects: handling missing values and tackling long-tailed distribution.

#### 3.4.1. Handling Missing Values

The missing values may affect the accuracy of trajectory prediction. Several studies [68,69] have proposed diverse solutions to tackle this problem. Qi et al. [70] employed an imitation learning paradigm with a variational autoencoder and a non-autoregressive transformation model. By jointly optimizing these two models, they predicted future trajectories and imputed missing values. Xu et al. [71] introduced a Multi-Space Graph Neural Network (MS-GNN) to extract compact spatial features from incomplete observation data. They also utilized a variational RNN to model temporal dependencies to learn missing patterns. On the other hand, Qin et al. [72] combined non-autoregressive and autoregressive approaches, effectively leveraging self-attention mechanisms at multiple levels to learn dependencies between observed and missing values. In general, the researchers used observed data to extrapolate missing values, mitigating their impact on trajectory predictions as much as possible. However, it is still an important development direction in pedestrian trajectory prediction to reduce the occurrence of missing values as much as possible through more reasonable observation methods.

#### 3.4.2. Tackling Long-Tailed Distribution

Most trajectories follow basic kinematic rules in real-world scenarios, while a minority exhibit complex motion patterns. Most pedestrians exhibit simple motion patterns, whereas a minority have more complex motion patterns. This situation is referred to as the long-tailed distribution of motion patterns. Due to the limited number of complex tail samples, models struggle to adequately learn their motion characteristics, leading to less accurate predictions on the tail samples.

Makansi et al. [73] first analyzed the long-tailed issue in pedestrian trajectory prediction. They observed that the features of tail samples were embedded between the features of head samples, causing the model to overlook the features of tail samples during training. To better extract features of tail samples, they introduced contrastive learning into trajectory prediction, which grouped similar sample features by distinguishing between head and tail samples. Zhou et al. [74] employed an ensemble network consisting of multiple prediction models to address the uncertainty brought by the long-tailed distribution. Subsequently, they devised a trajectory planner that considered the worst-case scenarios caused by prediction uncertainty, ensuring the safety of autonomous vehicles. Chen et al. [75] proposed a new sampling method called BOsampler, which used the initially predicted trajectories as prior conditions to explore low-probability potential paths adaptively. However, these two methods must fundamentally tackle the issue of long-tailed distribution. Recently, Wang et al. [76] introduced a plug-and-play trajectory prediction framework, dubbed FEND, which utilized future trajectory and employed contrastive learning to separate the trajectory samples of different motion patterns, thus forming independent clusters in the feature space. Building upon this, they introduced a distribution-aware hyper-predictor to learn the features of tail samples. The above methods can mitigate the long-tail issue but do not solve it. How to introduce more abundant trajectory data in diverse scenes and suppress the long-tail distribution from the perspective of data is also a task worth studying.

#### 3.4.3. Summary

The relevant methods for trajectory data issues are summarized in Table 5. Data-related issues are critical for data-driven pedestrian prediction methods. More new technologies must be introduced to solve these problems, especially for the mentioned issues of missing values and long-tail distribution.

**Suggestions:** The neural stochastic differential equation can be used to model the dynamic variation of the trajectory data, which is suitable for handling missing values caused by different factors. For the long-tail distribution issue, it is necessary to consider the diversity of head samples when designing the contrast learning framework to represent samples of different motion patterns more accurately. In addition, apart from the two data-related issues mentioned above, other data-related issues may need to be studied and solved.

**Possible limitations:** The methods mentioned above can only suppress the problems of missing data and long-tail distribution and cannot completely solve the problems existing in pedestrian trajectory data. In order to promote research in this field, it is also necessary to collect more high-quality, diversified, and multi-angle pedestrian trajectory data and solve data-related problems from the dataset’s construction itself.

### 3.5. Interpretability

Deep learning-based trajectory prediction methods use neural networks to model pedestrians’ motion patterns and forecast future trajectories. While they have achieved significant improvements in prediction accuracy compared to traditional methods, they still face the challenge of interpretability [77,78] due to the black-box characteristic of neural networks. Unlike neural networks, handcrafted methods attain interpretability by their rules and logic based on domain knowledge, expert experience, and specific logical inference. In addition, some classical machine learning methods can provide explainable trajectory prediction despite its low accuracy. Therefore, there are two ways to make deep learning-based trajectory prediction models interpretable: the combination with handcrafted methods and machine learning.

#### 3.5.1. Combination with Hand-Crafted Methods

Discrete choice models (DCMs) [79,80,81,82] are hand-crafted methods used to explain the decision-making behavior of pedestrians. While these models provide interpretable outputs, they often perform poorly regarding prediction accuracy. Therefore, most researchers combine DCMs with neural networks to tackle the interpretability issue in trajectory prediction. Kothari et al. [83] defined four pedestrian actions: keeping direction, leader-follower, collision avoidance, and occupancy. They employed a DCM to learn interpretable future intentions of pedestrians, which were further refined using neural networks. Several studies resorted to expert knowledge to obtain interpretability. For example, Cao et al. [84] introduced a knowledge-driven prediction approach based on logical information, which interpreted pedestrian behavior by learning spatiotemporal logical rules from observed trajectories. Neumeier et al. [85] incorporated expert knowledge into the decoder to generate an interpretable latent space for trajectory prediction.

#### 3.5.2. Combination with Machine Learning Methods

Some machine learning methods can model pedestrians’ motion patterns. Therefore, combining machine learning with neural networks can complement each other’s shortcomings. Shi et al. [86] applied decision trees to multi-modal trajectory prediction. Constructing a tree structure based on previously observed trajectory information and utilizing paths in the tree from the root to the leaf could describe pedestrians’ coarse-grained movement behaviors. Combining the interpretability of decision trees with the flexibility of neural networks enhances the performance and interpretability of trajectory prediction. Yue et al. [87] proposed BNSP-SFM, combining the behavioral stochastic differential equation (SDE) with Bayesian Neural Networks (BNNs). Neural networks contribute to excellent prediction capabilities, while SDE provides strong interpretability for the model.

#### 3.5.3. Summary

The relevant methods for model interpretability are summarized in Table 6. Interpretability is a crucial factor affecting the practical application of trajectory prediction models. Despite deep learning-based trajectory prediction methods have achieved high accuracy, the lack of interpretability makes them unsuitable for some security-related applications. Although existing studies have obtained interpretability by combining neural networks with machine learning or hand-crafted features, there are still shortcomings in prediction accuracy.

**Suggestions:** Future research on explainable trajectory prediction can focus on deep neural networks’ explainability. The Large Language Model (LLM) is also a significant way to provide interpretability of trajectory prediction. Considering the challenges of white-box neural networks or LLM-based trajectory prediction, interpretable trajectory predictions can now be obtained by generating some intermediate results, such as generating destinations, interactions between pedestrians, and pedestrian actions.

**Possible limitations:** LLMs are inevitably illusory and may give false interpretations. At the same time, the layout of LLMs will bring additional hardware costs and increase the overhead of the pedestrian trajectory prediction system.

## 4. Datasets and Evaluation Metrics

### 4.1. Evaluation Metrics

Mainstream evaluation metrics for pedestrian trajectory prediction consist of Average Displacement Error (ADE) and Final Displacement Error (FDE). ADE represents the average Euclidean distance between predicted and ground-truth trajectory coordinates within the prediction horizon. FDE denotes the Euclidean distance between predicted and ground-truth trajectory coordinates at the final time step. These two metrics are calculated as follows:

(2) $$\begin{array}{rrr} & & \;\mathrm{ADE}=\frac{\sum_{i=1}^{N}\sum_{t=1}^{T_{pred}}{∥{\hat{Y}}_{t}^{i}-Y_{t}^{i}∥}_{2}}{N\times T_{pred}}\\ & & \;\mathrm{FDE}=\frac{1}{N}\sum_{i=1}^{N}{∥{\hat{Y}}_{T_{pred}}^{i}-Y_{T_{pred}}^{i}∥}_{2} \end{array}$$

where *N* represents the pedestrian number; *T_pred_* and *T_obs_* represent the prediction and observation horizon; $\hat{Y}$ and *Y* represent the predicted and ground-truth trajectories, respectively; and ${∥\;∥}_{2}$ represents L2 norm.

### 4.2. Mainstream Pedestrian Trajectory Datasets

The mainstream pedestrian trajectory datasets contain rich scene information, covering pedestrian trajectories with complex interactive behaviors. These datasets provide a crucial foundation for the training and evaluating trajectory prediction models, helping them understand the motion patterns and rules in the real world. Here are some prevalent datasets:

(1) Stanford Drone [38]: The Stanford Drone Dataset (SDD) comprises trajectory data of pedestrians, cyclists, skateboarders, and vehicles captured using drones in 60 different scenes at Stanford University. SDD provides aerial images of these scenes and the pixel coordinates of agents in the scene. Additionally, the dataset includes various scene elements such as roads, sidewalks, buildings, parking lots, vegetation, and others.

SDD is commonly used for pedestrian trajectory prediction, and most studies adopt the split defined in TrajNet [88] for dividing the SDD into training, testing, and validation sets, as detailed in Table 7. In this split, the observation and prediction horizons are 8 time steps (3.2 s) and 12 time steps (4.8 s), respectively.

Table 8 reports the SOTA results on SDD. As reported in the table, MUSE-VAE [32] employed a multi-level prediction framework that spans from macroscopic to microscopic levels to address long-term trajectory prediction. In the macroscopic stage, the model learned a joint feature representation of the environment and agent motion to predict long-term and short-term motion goals. In the microscopic stage, it leveraged fine-grained spatiotemporal representations to predict the trajectories of agents. By combining the VAE framework with both macroscopic and microscopic stages, it considered joint uncertainties at two granularity levels, leading to promising results on the SDD. CSR [54], on the other hand, employed multiple non-shared CVAE models. These models predicted the next frame’s trajectory coordinates based on the initial historical observations, generating new historical observations, which were then fed into the next CVAE model until generating the complete trajectory. The design of such a cascaded CVAE model effectively avoided accumulating prediction errors, thus improving prediction performance. Additionally, CSR [54] enhanced interaction modeling and trajectory prediction compatibility by combining historical and predicted trajectory features globally.

(2) ETH [98] and UCY [99]: The ETH dataset consists of two scenes: ETH and Hotel. The UCY dataset comprises three scenes: UCY-ZARA1, UCY-ZARA2, and UCY-UNIV. All scenes were captured from a top-down perspective using surveillance cameras. ETH/UCY includes 1536 pedestrians and records thousands of non-linear trajectories. In these datasets, pedestrians exhibit complex behaviors, including movement from different directions, walking together, collision avoidance, and standing still. Like SDD, ETH/UCY is used to predict pedestrian trajectory. For data processing, the observed and predicted horizons are 8 time steps (3.2 s) and 12 time steps (4.8 s).

Table 9 reports the SOTA results on ETH/UCY. BNSP-SFM [87] and STGLow [96] achieved the best performance among them. The main advantage of BNSP-SFM [87] is its interpretability. It drew inspiration from Y-net [31] and decomposed the uncertainty into aleatoric uncertainty caused by behavior and epistemic uncertainty caused by unobservable cognitive factors. Specifically, BNSP-SFM modeled pedestrians’ behavioral dynamics as a second-order stochastic differential equation to handle aleatoric uncertainties arising from interactions. BNSP-SFM employed neural networks for epistemic uncertainty to predict pedestrians’ ultimate goals based on the scene and interactions. Additionally, to quantify the randomness of aleatoric uncertainties, they imposed priors on influencing factors and used Bayesian neural networks to study motion dynamics and uncertainties. In contrast to BNSP-SFM, STGLow [96] employed a flow-based generative model for prediction. Unlike GAN-based methods, flow-based approaches can directly model data in the latent space. CVAE-based methods optimize the variational lower bound of the log-likelihood of observed data, which may result in generated trajectories that do not align with reality. In contrast, flow-based methods can model data distribution more accurately, with clear physical meaning for simulating the evolution of human motion behavior. Additionally, STGLow combined GNNs with transformers to model temporal dependencies and spatial interactions fully, further enhancing prediction performance.

(3) NBA SportVU Dataset: The NBA trajectory dataset is collected by the NBA using the SportVU tracking system. This dataset provides the trajectories of 10 players and the basketball during actual games.

Table 10 summarizes the SOTA models on the NBA dataset. These models predict 20 future trajectories for 4.0 s using trajectories from the past 2.0 s. ADE and FDE are used to evaluate the prediction performance. Cao et al. [84] learned the spatiotemporal logical rules behind players’ behavior from a large amount of trajectory data using neural networks. Then, they used the learned spatiotemporal logical rules to infer the intentions of agents and optimize them using the expectation-maximization algorithm. Considering that the agents in the NBA dataset are athletes who adhere to specific behavioral rules, ref. [74] achieved promising performance on the NBA dataset. DynGroupNet [48] used dynamic multi-scale hypergraphs to model interactions between groups and capture the dynamic interactions of agents at different time steps through topological inference, neural message passing, and dynamic embedding evolution of multi-scale hypergraphs. It is worth noting that basketball players often need to cooperate to execute defensive or offensive strategies, making it a highly interactive sports scenario. Therefore, DynGroupNet [48] is well-suited to address such a dynamic interaction problem.

(4) JAAD [103] and PIE [104]: The JAAD dataset primarily records vehicle and pedestrian trajectories at intersections and provides a rich annotation collection extracted from over 240 h of driving footage, featuring 346 short video clips (5–10 s) that showcase typical urban driving scenarios under various weather conditions.

The PIE dataset includes recordings of typical traffic scenarios captured by onboard cameras, totaling 6 h. The dataset comprises 300,000 annotated video frames, 1842 pedestrian samples, and precise vehicle information from sensors, including speed, direction, and GPS coordinates, all synchronized with the video recordings.

A classic method on the JAAD/PIE datasets is SGNet [102], which maintains that goal information about the agents could provide more accurate and detailed predictions of future trajectories. However, unlike other studies that only consider the pedestrians’ final goals, SGNet [102] considered that pedestrians’ actions depend on the goals at different time scales. Therefore, it employed a lightweight module to estimate goal information at multiple time scales and enhance crucial goal features through an attention mechanism.

## 5. Conclusions and Prospects

This survey researches the challenges of deep learning-based pedestrian trajectory prediction and reviews existing solutions as well as mainstream datasets. The challenges faced by deep learning-based trajectory prediction methods are categorized into five aspects: motion uncertainty, interaction modeling, scene semantic understanding, data-related issues, and interpretability of prediction models. Subsequently, we summarize and analyze the solutions for each challenge, presenting prevalent trajectory prediction datasets alongside their SOTA methods. Inspired by the challenges of deep learning-based trajectory prediction methods, prospects can be explored in the following four aspects:

**(1)**  **Trajectory prediction for heterogeneous agents:** As mentioned in this survey, multi-agent interaction modeling is a significant challenge. Most current research needs to consider the impact of heterogeneous agents on interactions adequately. Predicting trajectories for heterogeneous agents is typically more difficult than for homogeneous agents. In real-world traffic scenarios, agents encompass various categories, such as pedestrians, cars, buses, and bicycles. For example, a pedestrian’s reaction when facing another pedestrian or a car can differ entirely. Even if a car has minimal impact on the target pedestrian, the target pedestrian may still react significantly. Some existing studies [42,97] have proposed solutions. Modeling heterogeneous agent interactions and performing trajectory prediction is a promising research direction.
**(2)**  **Lightweight trajectory prediction models:** Applying trajectory prediction in real-world scenarios necessitates balancing efficiency and accuracy. However, achieving this balance with deep learning-based trajectory prediction models can be challenging. The more factors a prediction model considers, the more accurate the predictions are. However, this increases the model’s complexity and reduces its efficiency. Furthermore, some prediction models based on memory-augmented networks [90,101] require a certain level of memory consumption, mainly when dealing with complex scenes. Therefore, designing lightweight network architectures that offer both efficiency and high accuracy is an important direction for future trajectory prediction research.
**(3)**  **Model robustness:** Trajectory prediction datasets used for training are often affected by noise and uncertainty. Additionally, in real-world applications, factors like sensor errors and weather introduce significant sources of uncertainty. In some real-world applications that emphasize security critically, the robustness against attacks has gained more and more attention. The attacks can occur at the training time by using approaches such as backdoor attacks to poison the training data. The attacker can also modify the training and inference procedures of a given model [105]. Therefore, it is crucial to prioritize model robustness to enhance the prediction model’s ability to withstand interference and improve its reliability, enabling it to be more effectively applied to various real-world tasks.
**(4)**  **Integration of trajectory prediction with motion planning:** Trajectory prediction is often used for downstream planning tasks. For instance, as demonstrated in ref. [94], a classification-based trajectory prediction approach was proposed, which assigned probabilities to predicted trajectories, thus better supporting downstream tasks. Therefore, exploring how to integrate trajectory prediction and motion planning tasks effectively is a valuable research task.

Although trajectory prediction has made commendable progress in recent years, there is still room for improvement. This survey will provide a better understanding of deep learning-based trajectory prediction for readers and the community and facilitate future research.

## Figures and Tables

**Table 1:** Comparison of several uncertainty prediction methods (results on the SDD [38]).

	Models	Year	ADE	FDE
Diversity Prediction	SocialVAE [24]	2022	8.10	11.72
	TDOR [25]	2022	6.77	10.46
Diffusion Models	MID [27]	2022	7.61	14.30
Goal Prediction	MUSE-VAE [32]	2022	6.36	11.10
	Goal-SAR [33]	2022	7.75	11.83

**Table 2:** Summary of methods for motion uncertainty.

Models	Year	Agents	Description
Social GAN [17]	2018	Pedestrians	Proposing diverse loss functions to generate multimodal trajectories
Social-BiGAT [16]	2019	Pedestrians	Generating reversible mapping between predictions and latent variables of motion
Social Ways [18]	2019	Pedestrians	Incorporating latent encoding into the random noise to enhance predictions
Y-net [31]	2020	Pedestrians	Modeling epistemic and aleatoric uncertainties by predicting goals and waypoints
DisDis [23]	2021	Pedestrians	Utilizing contrastive learning to categorize latent distributions
MG-GAN [20]	2021	Pedestrians	Dynamically selecting a suitable generator to avoid generating OOD samples
MUSE-VAE [32]	2022	Pedestrians	Predicting long- and short-term goals to mitigate uncertainty at two levels
Goal-SAR [33]	2022	Pedestrians	Integrating trajectory and goal information using a recurrent attention mechanism
SocialVAE [24]	2022	Pedestrians	Using FPC to enhance the diversity of trajectory predictions
SEEM [21]	2022	Pedestrians	Utilizing maximum sequence entropy as the loss function
TDOR [25]	2022	Pedestrians	Applying a symmetric cross-entropy loss to generate more realistic predictions
MID [27]	2022	Pedestrians	Gradually mitigating predicted uncertainty using a diffusion model
LED [29]	2023	Pedestrians	Introducing Leapfrog Initializer to speed up inference in the diffusion model
DACG [26]	2023	Pedestrians	Combining CVAE and GAN models for multimodal trajectory prediction
UPDD [30]	2023	Pedestrians	Parameterizing trajectories for improved diffusion model efficiency
ForceFormer [35]	2023	Pedestrians	Predicting goals based on observed trajectories and scene to guide agent motion

**Table 3:** Summary of methods for interaction modeling.

Models	Year	Agents	Interaction	Description
AgentFormer [50]	2021	Pedestrians	Transformer	Modeling interaction from temporal and social dimensions
DynGroupNet [48]	2022	Pedestrians	Hypergraph	Utilizing hypergraph for modeling dynamic interactions
CSR [54]	2022	Pedestrians	Social Pooling	Refining predictions by utilizing future interactions
STSF-Net [49]	2023	Pedestrians	SRM	Using SRM to capture spatiotemporal correlations
LSSTA [51]	2023	Pedestrians	GNN	Utilizing TCN for fusing spatiotemporal feature

**Table 4:** Summary of methods for scene understanding.

Models	Year	Agents	Description
Chen et al. [64]	2021	Pedestrians	Mitigating the effects of environmental bias using counterfactual analysis
Peng et al. [62]	2022	Pedestrians	Using CNN and attention mechanisms to extract and fuse scene information
IRLSOT [59]	2022	Pedestrians	Utilizing ENet to recognize scene and interacting with the scene through IRL
CTSGI [67]	2022	Pedestrians	Applying Do-calculus to mitigate the adverse effects of environmental bias
MGSU [60]	2023	Pedestrians	Using SegFormer and a multi-granularity fusion method to understand scene
SEAD [66]	2023	Pedestrians	Mitigating the effects of environmental bias using cross-attention mechanism

**Table 5:** Summary of methods for trajectory data issues.

Models	Year	Agents	Description
Qi et al. [70]	2020	Pedestrians	Using an imitation learning paradigm to address missing values
Makansi et al. [73]	2021	Pedestrians	Addressing long-tailed distribution through contrastive learning
FEND [76]	2023	Pedestrians	Proposing a future enhanced contrastive learning framework
GC-VRNN [71]	2023	Pedestrians	Introducing MS-GNN and RNN for addressing missing values
BOsampler [75]	2023	Pedestrians	A sampling method for exploring low-probability tail potential paths
INGRAIN [72]	2023	Pedestrians	Using ensemble learning to address long-tailed distribution

**Table 6:** Summary of methods for model interpretability.

Models	Year	Agents	Description
SAnchor [83]	2021	Pedestrians	Manually defining the motion rules for agents
SIT [86]	2022	Pedestrians	Using a decision tree to describe the motion of agents
Cao et al. [84]	2023	Pedestrians	Defining spatiotemporal logical rules for agents
BNSP-SFM [87]	2023	Pedestrians	Modeling the motion of agents using Bayesian SED

**Table 7:** Split of the SDD dataset.

Datasets	Scenes
Train	bookstore: 0–3; coupa: 3; deathCircle: 0–4; gates: 0, 1, 3–8; hyang: 4–7, 9; nexus: 0, 1, 3, 4, 7–9
Validation	bookstore: 4–6, coupa: 2; hyang: 2, 10–14; nexus: 2, 10, 11
Test	bookstore: 4–6; coupa: 2; hyang: 2, 10–14; nexus: 2, 10, 11

**Table 8:** SOTA results on SDD.

Models	Year	ADE	FDE
P2T [89]	2020	12.58	22.07
IRLSOT [59]	2022	9.66	13.05
SIT [86]	2022	9.13	15.42
MANTRA [90]	2021	8.96	17.76
LB-EBM [91]	2021	8.87	15.61
Graph-TERN [92]	2023	8.43	14.26
MSRL [93]	2023	8.22	13.39
SocialVAE [24]	2022	8.10	11.72
Y-net [31]	2020	7.85	11.85
Goal-SAR [33]	2022	7.75	11.83
PPNet [94]	2023	7.73	11.58
ExpertTraj [95]	2021	7.69	14.38
MID [27]	2022	7.61	14.30
STGLow [96]	2022	7.20	11.20
TDOR [25]	2022	6.77	10.46
E-V2-Net [97]	2023	6.57	10.49
NSP-SFM [37]	2022	6.52	10.61
BNSP-SFM [87]	2023	6.46	10.49
Cao et al. [84]	2023	6.41	10.23
MUSE-VAE [32]	2022	6.36	11.10
CSR [54]	2022	4.87	6.32

**Table 9:** SOTA results on ETH/UCY (ADE/FDE).

Models	Year	ETH	HOTEL	UNIV	ZARA1	ZARA2	AVG
MANTRA [90]	2021	0.48/0.88	0.17/0.33	0.37/0.81	0.27/0.58	0.30/0.67	0.32/0.65
UPDD [30]	2023	0.41/0.86	0.19/0.36	0.23/0.49	0.23/0.49	0.21/0.43	0.25/0.53
Graph-TERN [92]	2023	0.42/0.58	0.14/0.23	0.26/0.45	0.21/0.37	0.17/0.29	0.24/0.38
SIT [86]	2022	0.39/0.62	0.14/0.22	0.27/0.47	0.19/0.33	0.16/0.29	0.23/0.38
LSSTA [51]	2023	0.30/0.52	0.12/0.20	0.28/0.55	0.20/0.40	0.16/0.32	0.21/0.40
LB-EBM [91]	2021	0.30/0.52	0.13/0.20	0.27/0.52	0.20/0.37	0.15/0.29	0.21/0.38
MID [27]	2022	0.39/0.66	0.13/0.22	0.22/0.45	0.17/0.30	0.13/0.27	0.21/0.38
EgMotion [100]	2023	0.40/0.61	0.12/0.18	0.23/0.43	0.18/0.32	0.13/0.23	0.21/0.35
MemoNet [101]	2022	0.40/0.61	0.11/0.17	0.24/0.43	0.18/0.32	0.14/0.24	0.21/0.35
DACG [26]	2023	0.36/0.65	0.13/0.25	0.20/0.41	0.16/0.34	0.12/0.26	0.19/0.38
MSRL [93]	2023	0.28/0.47	0.14/0.22	0.24/0.43	0.17/0.30	0.14/0.23	0.19/0.33
ForceFomer [35]	2023	0.36/0.52	0.09/0.14	0.21/0.42	0.15/0.22	0.12/0.20	0.19/0.30
Goal-SAR [33]	2022	0.28/0.39	0.12/0.17	0.25/0.43	0.17/0.26	0.15/0.22	0.19/0.29
SGNet [102]	2022	0.35/0.65	0.12/0.24	0.20/0.42	0.12/0.24	0.10/0.21	0.18/0.35
AgentFormer [50]	2021	0.26/0.39	0.11/0.14	0.26/0.46	0.15/0.23	0.14/0.24	0.18/0.29
E-V2-Net [97]	2023	0.25/0.38	0.11/0.16	0.20/0.34	0.19/0.30	0.13/0.24	0.17/0.28
ExpertTrai [95]	2021	0.30/0.62	0.09/0.15	0.15/0.31	0.12/0.24	0.19/0.44	0.17/0.35
PPNet [94]	2023	0.22/0.33	0.09/0.13	0.22/0.34	0.18/0.27	0.14/0.22	0.17/0.25
BNSP-SFM [87]	2023	0.25/0.25	0.09/0.11	0.20/0.38	0.16/0.27	0.12/0.19	0.16/0.24
STGLow [96]	2022	0.31/0.40	0.09/0.14	0.16/0.33	0.12/0.24	0.09/0.19	0.15/0.28

**Table 10:** SOTA results on the NBA dataset (ADE/FDE).

Methods	Year	1.0 s	2.0 s	3.0 s	4.0 s
SocialVAE [24]	2022	0.49/0.66	0.77/1.11	1.11/1.46	1.37/1.79
MemoNet [101]	2022	0.38/0.56	0.71/1.14	1.00/1.57	1.25/1.47
Cao et al. [84]	2023	0.30/0.40	0.58/0.88	0.87/1.31	1.13/1.60
DynGroupNet [48]	2022	0.19/0.28	0.40/0.61	0.65/0.90	0.89/1.13
LED [29]	2023	0.18/0.27	0.37/0.56	0.58/0.84	0.81/1.10

## Data Availability

The raw data used to support the findings of this study are available from the corresponding author upon request.
